# Pregnancy-Associated Pyogenic Sacroiliitis: Case Report and Review

**DOI:** 10.1155/S1064744903000073

**Published:** 2003

**Authors:** Mohammad O. Almoujahed, Riad Khatib, Joseph Baran

**Affiliations:** Division of Infectious Diseases Department of Internal Medicine St John Hospital and Medical Center 22101 Moross Road Detroit MI 48236 USA

## Abstract

*Background*: Pyogenic sacroiliitis occurs infrequently during the peripartum period.

*Case:* A case at our institution and a review of the literature were analyzed. A total of 15 cases were discovered. The onset of illness was during pregnancy (40% of cases), within 3 weeks postpartum (40%) or post-abortion (20%), and the presentation was usually acute (< 7 days in 67% of cases). Frequent manifestations included localized pain in the hips or buttock, sacroiliac joint tenderness and fever. Computed tomography or magnetic resonance imaging revealed joint involvement in all cases tested. Microbiology was confirmed by blood (40%) or joint aspirate (75%), and most patients were treated with antibiotics. Surgical intervention took place in five cases. Pretermlabor was reported in only one case. All patients respondedwell to therapy without locomotive disability, and persistent pain was uncommon.

*Conclusion:* Septic sacroiliitis should be considered in peripartum patients who present with fever and severe localized pain. Medical management is usually curative, and without an adverse effect on pregnancy.


*Infect Dis Obstet Gynecol 2003;11:53–57*

### Pregnancy-associated pyogenic sacroiliitis: case report and review

**Mohammad O. Almoujahed, Riad Khatib and Joseph Baran**

*Division of Infectious Diseases, Department of Internal Medicine, St John Hospital and Medical Center, Detroit, MI*

***Background:*** Pyogenic sacroiliitis occurs infrequently during the peripartum period.
***Case:*** A case at our institution and a review of the literature were analyzed. A total of 15 cases were discovered. The onset of illness was during pregnancy (40% of cases), within 3 weeks postpartum (40%) or post-abortion (20%), and the presentation was usually acute (< 7 days in 67% of cases). Frequent manifestations included localized pain in the hips or buttock, sacroiliac joint tenderness and fever. Computed tomography or magnetic resonance imaging revealed joint involvement in all cases tested. Microbiology was confirmed by blood (40%) or joint aspirate (75%), and most patients were treated with antibiotics. Surgical intervention took place in five cases. Preterm labor was reported in only one case. All patients responded well to therapy without locomotive disability, and persistent pain was uncommon.
***Conclusion:*** Septic sacroiliitis should be considered in peripartum patients who present with fever and severe localized pain. Medical management is usually curative, and without an adverse effect on pregnancy.

Key words: SACROILIAC JOINT; SACROILIAC JOINT INFECTION; PERIPARTUM

### INTRODUCTION

Sacroiliac joint disease usually presents with low back pain that increases with ambulation. Although the majority of cases represent non-specific arthritis, this joint can be seeded after bacteremia, resulting in a pyogenic process[1]. This complication is more common in injection drug users, although it may develop after any bacteremia[1,2]. If it develops during pregnancy, it may pose a diagnostic challenge, as pain in the lower back and buttocks is common and often nonspecific during the pregnancy and postpartum periods[3,4]. We encountered such a case which prompted us to review all previously reported cases in the English literature and to present a comprehensive review of this subject in order to characterize the clinical manifestations, diagnostic approach, therapy and prognosis.

### CASE REPORT

A 26-year-old woman, gravida-1 (24 weeks' gestation), para-0, presented with severe pain in the right buttock region. This area was mildly

The findings described in this article were presented in part as Poster #22, Clinical Research 2001, American Federation for Medical Research, Arlington, Virginia, on 8 March 2001.

Correspondence to: Dr Joseph Baran Jr, Medical Education, St John Hospital, 22101 Moross Road, Detroit, MI 48236, USA. E-mail: joseph.baran@stjohn.org

© 2003 The Parthenon Publishing Group 

irritable for a few weeks, but the pain then became worse and began to radiate to the leg and to increase with ambulation. The patient denied experiencing feverishness, chills or other systemic symptoms. Her medical history was negative. She claimed that she did not smoke, drink alcohol or use injection drugs.

Physical examination was significant, revealing a temperature of 38.2°C and localized tenderness over the right sacroiliac joint. Laboratory tests revealed leukocytosis ($14.7 \times 10^6$ white blood cells/l with 87.7% neutrophils), an elevated erythrocyte sedimentation rate (70 mm/hour) and asymptomatic *E. coli* bacteriuria. Magnetic resonance imaging (MRI) of the lumbar spine was unrevealing. The patient was suspected of having either sacroiliitis or piriformis syndrome, and was started on oral prednisone, steroid injections in the right sacroiliac joint and cephalexin for the bacteriuria. She noted some relief of her shooting pain initially, but the remaining symptoms continued to worsen. MRI of the pelvis and sacroiliac joint was performed on day 11 after admission. It revealed widening of the right sacroiliac joint, a soft tissue density anterior and posterior to the joint space, and a fluid collection measuring $3 \times 3 \times 6$ cm within the iliacus muscle anterior to the right sacroiliac joint, consistent with sacroiliitis and a probable abscess.

A computed tomography-guided aspiration of the sacroiliac joint fluid collection yielded 2.2 ml of thick, brownish, chocolate-like fluid, and the Gram stain showed Gram-positive cocci in clusters. The patient was started on cefazolin IV 6 grams/day, and steroids were gradually discontinued. Culture grew methicillin-susceptible *Staphylococcus aureus*. The patient showed clinical and radiological improvement with follow-up MRI. Cefazolin was continued for a 6-week course. She subsequently had an uneventful normal vaginal delivery. At a follow-up visit 6 months after discharge, she was doing well with normal ambulation, although she continued to have mild discomfort in the right buttock.

### SUBJECTS AND METHODS

A total of 14 cases published in English were identified through a MEDLINE search for articles (1966–2001) and relevant bibliographies. Search terms that were used included septic sacroiliitis, sacroiliitis, sacroiliac joint, septic arthritis and pregnancy. All cases identified in addition to ours were included in the review[1,3–12]. Demographics, risk factors, clinical manifestations, microbiology, radiological studies, management and outcome were noted.

### RESULTS

The mean age of the patients was 25.4 years (range 17–35 years). The onset of illness was during pregnancy in six cases (40%)[1,4,6,10,11] (including this case) within 3 weeks of delivery in six instances (40%)[3,5,7–9] and within 3 weeks of abortion in three cases (20%)[7,11,12]. Among the postpartum cases, forceps outlet delivery was utilized in one case, 9 days prior to presentation[9]. In addition, delivery was complicated by an episode of chills and bleeding within 24 hours of vaginal delivery in one case[5], and by high fever with disseminated intravascular coagulopathy and fetal distress in another case[5]. Among the three post-abortion cases, termination of pregnancy was spontaneous in one case[12], induced but incomplete in one case[11], and followed induction due to fetal death in the other case[7].

Possible risk factors were identified in eight patients (53.3%), including injection drug use ($n = 3$; 20%)[1,3,10], infective endocarditis ($n = 2$; 13%)[4,10], urinary tract infection ($n = 3$; 20%)[1,6,10], endometritis ($n = 2$; 13%)[5,11] and extensive sinusitis requiring multiple irrigations in the pre-antibiotic era[11].

The clinical characteristics are listed in Table 1. Frequent manifestations included localized pain (100% of cases), sacroiliac joint tenderness (80%) and ambulatory impairment (47%). The interval between the onset of symptoms and diagnosis was 2–32 days, although the majority of cases (66.7%) had an acute onset (< 7 days). Of interest, fever was absent in a substantial number of cases ($n = 5$; 33.3% of patients with an adequate history)[1,3,6,10,12]. High fever and extension of infection to the surrounding structure were common in patients reported in the pre-antibiotic era, presumably because of a delay in recognition and lack of therapy[11,12]. Leukocytosis was present in eight

**Table 1** Clinical characteristics of pyogenic sacroiliitis associated with pregnancy or the postnatal period

| | *Age* | | | | | *Culture*[c] | | *Treatment* | |
|---|---|---|---|---|---|---|---|---|---|
| *Reference* | *Years (time)*[a] | *Risk factors*[b] | *Onset* | *Symptoms* | *Organism* | *BC* | *Joint* | *Antibiotics*[d] | *Surgery* |
| 1 | 33 (25 weeks) | IDU, UTI | Acute (3 days) | Buttock pain | *Staphylococcus epidermidis* | – | + | Nafcillin (42 days) | Open drainage |
| 3 | 23 (1 day) (post-P) | IDU | Subacute (21 days) | Back and buttock pain | *Staphylococcus aureus* | – | + | Nafcillin (42 days) | None |
| 4 | 24 (26 weeks) | Endocarditis | Acute (1 day) | Back and hip pain; fever | Alpha *Streptococcus* | + | ND | Not stated (42 days) | None |
| 5 | 26 (3 weeks) (post-P) | Endometritis | Subacute (21 days) | Buttock pain; fever | Not defined | – | ND | Ceftriaxone (42 days) | None |
| 5 | 26 (3 weeks) (post-P) | None | Subacute (21 days) | Buttock pain; fever | Not defined | ND | ND | Pris/Rif (30 days) | None |
| 6 | 18 (28 weeks) | UTI | Acute (6 days) | Hip pain | *Staphylococcus species* | ND | + | Flufloxacin | Open drainage |
| 7 | 35 (at abortion) | None | Acute | Buttock pain; fever | *Staphylococcus aureus* | + | ND | Not stated | None |
| 7 | 23 (2 weeks) (post-P) | None | Acute | Buttock pain; fever | Not defined | – | ND | Not stated (56 days) | None |
| 8 | 32 (2 days) (post-P) | None | Acute (1 day) | Buttock pain; fever | Not defined | – | – | Dicloxacillin, ceftri-axone (60 days) | None |
| 9 | 26 (9 days) (post-P) | None | Acute (4 days) | Buttock pain; fever | Group B *Streptococcus* | + | ND | Not stated (42 days) | None |
| 10 | 17 (24 weeks) | IDU, UTI, endocarditis | Acute (5 days) | Back and buttock pain | *Staphylococcus aureus* | + | – | Not stated (49 days) | None |
| 11 | 20 (2 weeks) (post-A) | Endometritis | Acute (2 days) | Leg pain; fever | *Streptococcus pneumoniae* | ND | + | Not stated | Resection |
| 11 | 28 (32 weeks) | Sinusitis | Subacute | Back pain; fever | *Streptococcus pneumoniae* | – | + | Not stated | Resection |
| 12 | 24 (3 weeks) (post-A) | None | Acute (7 days) | Back pain | Not defined | ND | ND | Not stated | Debridement |
| Case | 26 (24 weeks) | None | Subacute (14 days) | Buttock pain; fever | *Staphylococcus aureus* | ND | + | Cefazolin (42 days) | None |

[a]Post-P, postpartum; post-A, post-abortion; [b]IDU, injection drug use; UTI, urinary tract infection; [c]–, negative; +, positive; ND, not done; [d]Pris, pristinamycin; Rif, rifampin of 13 cases (62%), and an elevated erythrocyte sedimentation rate was observed in eight of eight patients (100%).

Routine X-rays often yielded nonspecific findings, whereas computed tomography (CT) and MRI, which were performed in seven and six cases, respectively, showed evidence of bone erosion and joint involvement in all cases tested. A bone scan was obtained in five postpartum or post-abortion cases, and it demonstrated increased localized isotope uptake in the involved joint, consistent with sacroiliitis, in all cases. Bilateral involvement was uncommon ($n = 2$; 13.3%).

Microbiological etiology was determined in 66.7% of cases. Bacteremia was documented in four of ten cases and joint fluid culture was positive in six of eight cases. In five cases, no microorganism was identified. The most common organism was *Staphylococcus aureus*.

The management was described in 14 cases. Three cases were reported in the pre-antibiotic era[11,12], all of which were treated with surgical debridement. The remaining cases received intravenous antibiotics for 30–60 days; the median period was 6 weeks. Surgical drainage was required in only two cases[1,6]. All of the patients recovered, and only two patients had residual pain. Preterm labor was reported in one case[3].

### DISCUSSION

The diagnosis of pathologic conditions in the sacroiliac joints during pregnancy may pose a diagnostic challenge. Low back pain, which is the usual presentation of inflammatory processes in this joint, is fairly common during pregnancy. Furthermore, the rare incidence of the disease, the nonspecific symptoms and the difficulty in localizing the pain without careful examination all contribute to the problem of establishing the diagnosis. Therefore when pregnant women develop septic sacroiliitis, the initial manifestations can be attributed to pregnancy-associated arthropathy. The presenting manifestations are still nonspecific and fever is absent in a substantial number of patients. Our case illustrates this challenge. This patient presented with increasing backache that was initially attributed to nerve compression. The sacroiliac source was not suspected for several days.

Our review illustrates the fact that this condition is relatively rare. In a comprehensive review of all relevant publications from 1878 to 1990, Vyskocil *et al.*[1] identified 163 cases of pyogenic sacroiliitis, only four of which were associated with pregnancy[1]. Since then, we have found ten additional cases[3–12]. Our findings demonstrate that without proper diagnosis and treatment, the infection frequently extends to the periarticular structures[11,12]. In addition, in cases that involved preterm labor or fetal death, it is uncertain whether joint infection preceded, coincided with or followed these obstetric events[3,7,11].

The pathogenesis of the condition is suspected to be hematogenous. In support of this conclusion, this condition was seen either in patients with a history of injection drug use or in those who had recently had an infection elsewhere, including endocarditis, sinusitis and urinary tract infection. The most common organism was *Staphylococcus aureus*.

With regard to diagnosis, routine X-ray was rarely helpful. Bone scan was helpful, but it should only be done during the postpartum period because of concerns about radiation exposure. CT and MRI are both very helpful. MRI is probably the method of choice in pregnancy, as it provides a highly sensitive means of detailed evaluation of the joint and surrounding soft tissue without exposing the fetus to ionizing radiation[4].

The treatment for pregnancy-related bacterial sacroiliitis is similar to that for nonpregnancy-related cases. Most authors recommend 4–6 weeks of parenteral antibiotic therapy. Only two patients required surgical drainage after initiating antibiotics, and in both cases the disease extended to the surrounding structure[1,6]. The outcome of treatment appears to be favorable.

### REFERENCES

1. Vyskocil J, McIlroy M, Brennan T, *et al.* Pyogenic infection of the sacroiliac joint: case report and review of the literature. *Medicine* 1991;70:188–97
2. Zimmermann B III, Mikolich DJ, Lally EV. Septic sacroiliitis. *Semin Arthritis Rheum* 1996;26:592–604
3. Gordon G, Kabins SA. Pyogenic sacroiliitis. *Am J Med* 1980;69:50–6
4. Wilbur A, Langer B, Spigos D. Diagnosis of sacroiliac joint infection in pregnancy by magnetic resonance imaging. *Magn Reson Imaging* 1998; 6:341–3
5. Tisserant R, Loeuille D, Pere P, *et al.* Septic sacroiliitis during the postpartal period: diagnostic contribution of magnetic resonance imaging. *Rev Rhum* 1999;66:512–15
6. Sandrasegaran K, Saifudin A, Coral A, *et al.* Magnetic resonance imaging of septic sacroiliitis. *Skeletal Radiol* 1994;23:289–92
7. Siam A, Hammoudeh M, Uwaydah A. Pyogenic sacroiliitis in Qatar. *Br J Rheumatol* 1993;32: 699–701
8. Linnet K, Kammelgaard L, Johansen M, *et al.* Bilateral pyogenic sacroiliitis following uncomplicated pregnancy and labor. *Acta Obstet Gynecol Scand* 1996;75:950–1
9. Jedwab M, Ovadia S, Dan M. Pyogenic sacroiliitis in pregnancy. *Int J Gynaecol Obstet* 1999;65:303–4
10. Egerman RS, Mabie WC, Eifrid M, *et al.* Sacroiliitis associated with pyelonephritis in pregnancy. *Obstet Gynecol* 1995;85:834–5
11. Chandler F. Pneumococcic infection of the sacroiliac joint complicating pregnancy. *JAMA* 1933;101:114–16
12. L'Episcopo JB. Suppurative arthritis of the sacroiliac joint. *Ann Surg* 1936;104:289–303

Received 07/16/02; Accepted 10/08/02
